# ID 139 - Influência da Variação da Taxa de Desconto em Avaliações Econômicas em Saúde

**DOI:** 10.5327/2237-9622.2025.v34s1.139

**Published:** 2025-11-25

**Authors:** Mariana Millan Fachi, Vinicius Lins Ferreira, Ludmila Peres Gargano, Haliton Alves Oliveira, Rosa Camila Lucchetta, Layssa Andrade Oliveira

**Keywords:** avaliação econômica em saúde, taxa de desconto, razão custo-efetividade incremental

## Abstract

**Introdução:**

A taxa de desconto (TD) é um elemento crucial nas avaliações econômicas (AE), pois permite ajustar custos e benefícios futuros ao seu valor presente, refletindo a preferência temporal dos recursos. Embora a TD possa ser percebida como uma abordagem meramente técnica, apresenta variações (1,5% a 10% ao ano), a depender do país. O valor adotado por cada país tende a refletir o ajuste do valor futuro para o presente, sendo que em países com elevadas taxas de inflação, a TD tende a ser de ≥5%. Sabe-se que a ampliação da TD é mais recomendada para AE com horizontes temporais (HT) longos, sem uma delimitação temporal. Devido às incertezas relacionados ao valor a ser adotado e quando aplicar a TD, este trabalho visa compreender a influência da TD em AE conduzidas para o SUS.

**Método:**

Foi conduzida uma análise retrospectiva com base em 41 modelos econômicos realizados pelos autores em 2023 e 2024, variando a TD entre 0% e 10% e avaliando a influência na razão custo-efetividade incremental (RCEI) (Análise 1). Ainda, foram analisados 87 relatórios publicados em 2023 e 2024 no site da Conitec, para identificar a aplicação da TD (análise 2).

**Resultados:**

Na análise 1, 33 (80,5%) AEs avaliadas eram de tecnologias com indicações oncológicas. O HT mediano foi de 20 anos (intervalo interquartil [IIQ]: 10-30). Ao testar a TD de 0%, 3% e 10%, o valor de RCEI para AV ganho variou de -56% a 3%, -26% a 1% e -2% a 87%, respectivamente. Já para QALY ganho, o RCEI variou entre -51% e 70%, -23% e 26% e -56% e 72%, respectivamente. Duas tecnologias avaliadas tiveram mudança em relação ao limiar estabelecido pela Conitec, estando abaixo deste para AV ganho (TD 5% de R$ 50.712 para TD 0% de R$ 34.170) e QALY ganho (TD 5% de 43.191 para TD 10% de R$ 31.432). Uma tecnologia mudou a classificação quando considerado três vezes o limiar estabelecido para QALY ganho (TD 5% de R$ 112.273 para TD 10% de R$ 133.330). Na análise 2, 54 (62,1%) relatórios aplicaram a TD, sendo que 53 usaram conforme recomendação da diretriz (5%). Destes, 30 não deixaram claro se testaram este parâmetro na análise de sensibilidade, enquanto os demais testaram as seguintes TD: 0% e 10% (n=12), 3% e 6% (n=1), 4% e 6% (n=3), 4,5% e 5,5% (n=3), 3,5% (n=1) e 0% (n=3). Dos 14 relatórios que foi possível extrair os dados dessa análise de sensibilidade, nenhum resultado mudou a classificação com relação ao limiar.

**Conclusão:**

A análise de sensibilidade da TD é uma etapa essencial em AE, mesmo quando os resultados iniciais não indicam mudanças significativas com diferentes TD. Isso se justifica pelas fragilidades e incertezas inerentes aos modelos econômicos e pela importância de garantir que as conclusões sejam robustas frente a variações em parâmetros críticos. Neste estudo, a aplicação de diferentes TD revelou flutuações importantes no valor da RCEI, sendo que a TD de 10% gerou maiores variações, especialmente em análises de longo HT. Apesar da baixa frequência com que as conclusões de custo-efetividade foram alteradas nesta análise, conclui-se que a avaliação da influência da TD permanece sendo importante para subsidiar a tomada de decisão. É importante considerar que a maioria das análises eram do escopo da oncologia, em que, em geral, os HT são longos e os custos e benefícios são diluídos neste período. Esta análise não contemplou tecnologias cujo impacto econômico e clínico seja imediato, ainda que em HT longos, em que a redução da TD pode ser ainda mais influente nos resultados.

